# Novel *TTLL5* Variants Associated with Cone-Rod Dystrophy and Early-Onset Severe Retinal Dystrophy

**DOI:** 10.3390/ijms22126410

**Published:** 2021-06-15

**Authors:** Vasily Smirnov, Olivier Grunewald, Jean Muller, Christina Zeitz, Carolin D. Obermaier, Aurore Devos, Valérie Pelletier, Béatrice Bocquet, Camille Andrieu, Jean-Louis Bacquet, Elodie Lebredonchel, Saddek Mohand-Saïd, Sabine Defoort-Dhellemmes, José-Alain Sahel, Hélène Dollfus, Xavier Zanlonghi, Isabelle Audo, Isabelle Meunier, Elise Boulanger-Scemama, Claire-Marie Dhaenens

**Affiliations:** 1 Université de Lille, Faculté de Médecine, 59037 Lille, France; vasily.smirnov@chru-lille.fr; 2 CHU Lille, Service d’Exploration Fonctionnelle de la Vision et de Neuro-Ophtalmologie, Hôpital Salengro, 59037 Lille, France; sabine.defoort@chru-lille.fr; 3 Sorbonne Université, INSERM, CNRS, Institut de la Vision, 75012 Paris, France; christina.zeitz@inserm.fr (C.Z.); saddekms@gmail.com (S.M.-S.); j.sahel@gmail.com (J.-A.S.); isabelle.audo@inserm.fr (I.A.); 4 Univ. Lille, Inserm, CHU Lille, U1172-LilNCog-Lille Neuroscience & Cognition, 59045 Lille, France; olivier.grunewald@chru-lille.fr; 5 Laboratoire de Génétique Médicale, Institut de Génétique Médicale d’Alsace (IGMA), INSERM U1112, Fédération de Médecine Translationnelle de Strasbourg (FMTS), Université de Strasbourg UMRS_1112, 67000 Strasbourg, France; jeanmuller@unistra.fr; 6 Laboratoire de Diagnostic Génétique, Hôpitaux Universitaires de Strasbourg, Institut de Génétique Médicale d’Alsace (IGMA), 67000 Strasbourg, France; 7 Praxis für Humangenetik Tuebingen & Center for Genomics and Transcriptomics, CeGaT GmbH, 72076 Tuebingen, Germany; Carolin.Obermaier@humangenetik-tuebingen.de; 8 Univ. Lille, CHU Lille, Service de Toxicologie et Génopathies, 59037 Lille, France; aurore.devos@chru-lille.fr (A.D.); elodie.lebredonchel@chru-lille.fr (E.L.); 9 Centre de Référence pour les Affections Rares en Génétique Ophtalmologiques, Hopitaux Universitaires de Strasbourg, 67000 Strasbourg, France; valerie.pelletier@chru-strasbourg.fr (V.P.); jean-louis.bacquet@chru-strasbourg.fr (J.-L.B.); dollfus@unistra.fr (H.D.); 10 National Reference Centre for Inherited Sensory Diseases, University of Montpellier, Montpellier University Hospital, Sensgene Care Network, ERN-EYE Network, 34295 Montpellier, France; beatrice.bocquet@inserm.fr (B.B.); isabelannemeunier@yahoo.fr (I.M.); 11 Institute for Neurosciences of Montpellier (INM), INSERM, University of Montpellier, INSERM, 34295 Montpellier, France; 12 Centre Hospitalier National d’Ophtalmologie des Quinze-Vingts, INSERM-DHOS CIC 1423, 75012 Paris, France; candrieu@for.paris; 13 Fondation Ophtalmologique Adolphe de Rothschild, 75019 Paris, France; eboulanger@for.paris; 14 Department of Ophthalmology, The University of Pittsburgh School of Medicine, Pittsburgh, PA 15213, USA; 15 Service d’Ophtalmologie, CHU de Rennes, 35000 Rennes, France; dr.zanlonghi@gmail.com; 16 Institute of Ophthalmology, University College London, London EC1V 9EL, UK

**Keywords:** *TTLL5* gene, novel variants, large deletion, cone-rod dystrophy, early onset severe retinal dystrophy

## Abstract

Variants of the *TTLL5* gene, which encodes tubulin tyrosine ligase-like family member five, are a rare cause of cone dystrophy (COD) or cone-rod dystrophy (CORD). To date, only a few *TTLL5* patients have been clinically and genetically described. In this study, we report five patients harbouring biallelic variants of *TTLL5.* Four adult patients presented either COD or CORD with onset in the late teenage years. The youngest patient had a phenotype of early onset severe retinal dystrophy (EOSRD). Genetic analysis was performed by targeted next generation sequencing of gene panels and assessment of copy number variants (CNV). We identified eight variants, of which six were novel, including two large multiexon deletions in patients with COD or CORD, while the EOSRD patient harboured the novel homozygous p.(Trp640*) variant and three distinct *USH2A* variants, which might explain the observed rod involvement. Our study highlights the role of *TTLL5* in COD/CORD and the importance of large deletions. These findings suggest that COD or CORD patients lacking variants in known genes may harbour CNVs to be discovered in *TTLL5,* previously undetected by classical sequencing methods. In addition, variable phenotypes in *TTLL5*-associated patients might be due to the presence of additional gene defects.

## 1. Introduction

Cone and cone-rod dystrophies constitute a heterogeneous group of inherited retinal disorders primarily affecting cone photoreceptors. In cone dystrophies (COD), alterations are generally restricted to the cones, but can extend to the rod photoreceptors at a later stage [1]. COD begins in late childhood or early adult life with visual loss, abnormal colour vision, photophobia and central scotoma [2]. The fundus is variably altered, ranging from no alteration to bull’s eye maculopathy. The retinal periphery is usually preserved. The optic discs show a variable degree of temporal pallor [2]. Cases of pure COD are very rare. Usually, there is a clinical continuum between COD and cone-rod dystrophies (CORD). The course of CORD is more severe than that of COD, but both disorders progress to complete blindness in most cases [3].

The estimated prevalence for nonsyndromic COD and CORD is between one in 30,000 and one in 40,000 [4], and all modes of Mendelian inheritance have been reported. They are highly genetically heterogeneous, but as COD and CORD overlap strongly in their clinical characteristics, the genetic descriptions of both entities are generally merged. Ten genes involved in autosomal dominant forms of COD/CORD, twenty-three genes involved in recessive forms and two genes involved in X-linked forms have been reported in the Retnet database (https://sph.uth.edu/retnet/sum-dis.htm#B-diseases, 21 January 2021 version). However, 30–50% of recessive or simplex COD/CORD cases remain unsolved [5,6,7].

In 2014, a new gene, *TTLL5* (MIM 612268, RefSeq accession number NM_015072.4), which encodes tubulin tyrosine ligase-like family member five, was described in COD/CORD patients [8]. *TTLL5* contains 32 exons, among which 31 encode a protein of 1281 amino acid residues. *TTLL5* is highly expressed in the brain, testis, and retina [8,9,10] and is mainly involved in polyglutamylation, a posttranslational modification consisting of the addition of glutamic acids to a glutamate residue on targeted proteins within the primary cilium. Moreover, it glutamylates the glutamic acid and glycine repeated residues of RPGR^ORF15^ [11,12]. In the human retina, *TTLL5* is mainly expressed in cones and at lower levels in rod photoreceptors [8]. 

Only 17 patients from 14 families with COD/CORD associated with *TTLL5* gene defects have been described to date, and among these patients, 14 have been clinically detailed [8,13,14,15,16,17]. Here, we report a complete clinical description of five French patients presenting either COD/CORD or early-onset severe retinal dystrophy (EOSRD) linked to six novel pathogenic biallelic variants, among which two are large intragenic deletions in *TTLL5*.

## 2. Results

### 2.1. Phenotypic Characterization

Here, we describe five individuals from five families with biallelic pathogenic variants in the TTLL5 gene, four patients with teenage onset COD/CORD and one patient (IA-CIC08269) with EOSRD. A summary of the clinical data is presented in Table 1.

**Figure 1:**
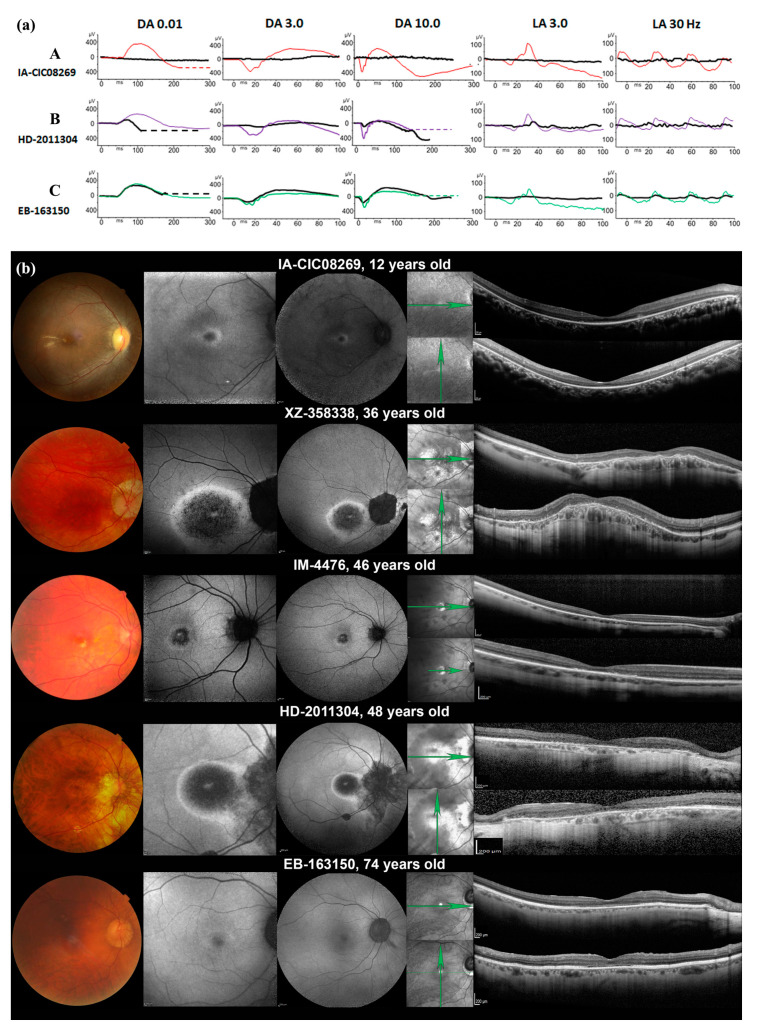
Full-field ISCEV-standard ERGs and Retinal imaging data. (**a**) In bold black, patients’ ffERGs. In thin colour, age- and sex-matched controls. A. Early-onset severe retinal dystrophy. subject IA-CIC08269, 7 y.o. Undetectable dark- and light-adapted responses. B. Cone-rod dystrophy. subject HD-2011304, 48 y.o., mild reduction of dark-adapted responses (DA 0.01, DA 3.0), more pronounced at higher intensity flash (DA 10.0). Severe reduction of light-adapted responses and marked implicit time delay. C. Cone dystrophy. Subject EB-163150, 72 y.o. Normal dark-adapted responses to dim (DA 0.01) and standard (DA 3.0) flash, small a-wave reduction to bright flash (DA 10.0). Residual light-adapted responses; (**b**) Only right eyes are shown. Columns (in order): fundus photography, SWAF 30°, SWAF 55°, IRR (the green arrows indicate the orientation of OCT scans), SD-OCT. Subject IA-CIC08269 presents with optic disc pallor, vascular attenuation, pigmentary changes of the fovea and outside the vascular arcades with yellowish band-like changes near the fovea. SWAF reveals a small perifoveal ring of increased autofluorescence. SD-OCT shows a preservation of the hyper reflective outer retinal bands at the fovea with outer nuclear layer thinning, some paramacular hyperreflective subretinal dots (better seen on the horizontal scan) with major thinning of the outer retina in the periphery. Subject XZ-358338 presents with some arteriolar narrowing, peripapillary chorioretinal atrophy and pigmentary changes in the macula. SWAF revealed a central area of loss of autofluorescence surrounded by a ring of increased autofluorescence leading to a bull’s eye appearance. SD-OCT shows a dome-shaped macula with disorganized hyper reflective outer retinal bands and severe thinning of the ONL while the outer retina is preserved in the periphery. IM-4476 has normal optic disc and retinal vessels; a small foveal lesion surrounded by yellowish material. SWAF reveal a foveal loss of autofluorescence outlined by an irregular ring of increased autofluorescence. SD-OCT shows a global thinning of the ONL, disorganized outer retinal hyper-reflective bands and some hyper-reflective dots in the parafoveal region. Subject HD-2011304 has optic disc pallor, arteriolar narrowing, peripapillary and, perifoveolar chorioretinal atrophy. SWAF shows a central hyper autofluorescent spot surrounded by an area of autofluorescent loss outlined by a ring of increased autofluorescence. SD-OCT revealed foveal sparing surrounded by a loss of the outer retinal layers which reappear in the periphery. Subject EB-163150′s fundus shows vascular narrowing and subtle foveal change. SWAF shows a subtle perifoveal ring of increased autofluorescence. SD-OCT shows a focal loss of the EZ and interdigitation zone at the fovea leading to a cavitation.

Subject IA-CIC08269 was the second child of consanguineous parents (first-degree cousins). She had poor vision since early childhood and night blindness since 6 y.o. BCVA for both eyes was reduced to 20/50 at 7 y.o. The visual field to the III1e target was constricted to 5°, while target I1e was not perceived. There was a tritan axis colour vision defect. The ffERG response was undetectable under both scotopic and photopic conditions (Figure 1a(A)). Fundus examination revealed mild narrowing of the retinal vasculature and some pigmentary changes around the fovea and outside the vascular arcades (Figure 1b). Only a small foveal island of the outer retinal layers (i.e., external limiting membrane (ELM) and ellipsoid zone (EZ)) was preserved on SD-OCT, with thinning of the outer nuclear layer (ONL). These foveal changes were correlated with a small perifoveal ring of increased autofluorescence on SWAF. The patient had been tested for hearing impairment at ages 8 and 9, and both audiograms were normal.

Four subjects (XZ-358338, IM-4476, HD-2011304 and EB-163150) shared nearly the same clinical presentation (Table 1 and Figure 1b). These patients reported poor visual acuity from their teenage years. Photophobia appeared in their third or fourth decade. They were all highly myopic. Visual field testing revealed absolute or relative central scotomata of small size (5° to 15°) and a preserved peripheral visual field. Colour vision testing revealed a tritan axis in all but one patient (IM-4476), who presented with severe dyschromatopsia. The ffERG was consistent with pure cone dysfunction in patients IM-4476 and EB-163150; combined cone-rod dysfunction was found in subjects XZ-358338 and HD-2011304 (Figure 1a (B,C)). Cataracts were observed in the oldest subject (EB-163150), and patient XZ-358338 had a phakic intraocular lens for myopia correction. Regarding the fundus aspects, all presented a depigmented round macular lesion with more or less distinct edges (bull’s eye maculopathy), and all had myopic fundus changes (peripapillary atrophy and chorioretinal thinning in the posterior pole, Figure 1b. SWAF showed a round macular lesion of decreased autofluorescence with hyperautofluorescent edges and a small hyperautofluorescent foveal dot in subjects XZ-358338, IM-4476 and HD-2011304; subject EB-163150 had a dark fovea circled with an ill-limited ring of increased autofluorescence. NIR images showed an increased reflectance corresponding to the macular lesions. SD-OCT scans revealed various degrees of outer retinal (ONL and EZ) and RPE hyporeflectivity within the posterior pole. Subject XZ-358338 had a dome-shaped macula.

Two of the four adult patients had children (Supplementary Figure S1). For the other two, the absence of offspring was not investigated. It is therefore not known whether infertility was the cause.

### 2.2. Genotyping Results

Overall, we identified eight variations, of which six were novel, encompassing three missense and two nonsense variants, one splice site variant and two large multiexon deletions in *TTLL5* (Table 2). All were classified as pathogenic or likely pathogenic according to the ACMG (American College of Medical Genetics and Genomics) criteria [18].

**Figure 2:**
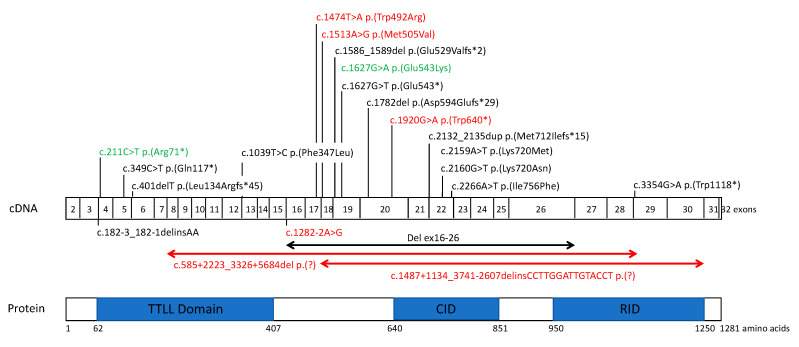
Localization of all reported and novel *TTLL5* variants in cone and cone-rod dystrophies. Novel variations are in red, previously reported variants are in black and variants previously reported found in this study are in green. cDNA and protein (if known) nomenclatures are reported for each variant. *TTLL5* is composed of 32 exons (depicted as boxes) and encodes 1281 amino acids. Protein domains, presented in blue boxes in the lower part, are as follows: TTLL core domain (delineated from amino acid 62 to 407) has a role in side chain elongation activity and in initiating the side chain, and polyglutamylation activity requires addition sequences of 100–150 amino acids on either side of the core TTL domain for full activity; c-MTBD (cationic microtubule binding domain) (amino acids 378 to 488); CID (cofactor interaction domain) (amino acids 640 to 841); RID (receptor interaction domain) has an unknown function, a C-terminal extension, and is required for transcription factor activity.

Among the single nucleotide variants, c.211C>T p.(Arg71*) was recently reported [17], and c.1920G>A p.(Trp640*) is novel. They are located in exon 4 (TTLL domain) and exon 20 (CID domain) (Figure 2). Both variants are rare. Indeed, p.(Arg71*) is present in 0.00002% of the non-Finnish European population, while p.(Trp640*) has not been reported in gnomAD V2.1.1. 

Patient IA-CIC08269 harboured a presumably homozygous c.1920G>A p.(Trp640*) nonsense variant in *TTLL5*, since no copy number variant was found. Three additional heterozygous variants were identified in *USH2A*: two missense variants, c.4586A>T p.(Lys1529Ile) and c.6800C>T p.(Pro2267Leu), and one splice variant, c.15297+3A>G p.(?). The p.(Lys1529Ile) variant [19] is considered to be of unknown significance according to ACMG criteria, whereas the not yet reported p.(Pro2267Leu) is classified as likely pathogenic. Loss of the splice donor site was predicted for the c.15297+3A>G variant (SpiceAI score: 0.65; MaxEnt score: −42.1%). However, it was classified as a VUS in LOVD and in ClinVar. Unfortunately, cosegregation analysis could not be performed due to the lack of parental DNA samples. 

We also identified two novel missense variants, c.1474T>A, p.(Trp492Arg) and c.1513A>G, p.(Met505Val), in exons 17 and 18, respectively, located in the region surrounded by the TTLL and CID domains. These variants are rare: the first variant has not been reported, and the second is found with a 0.00005% frequency in the non-Finnish European population in gnomAD. The p.(Trp492Arg) variant is highly conserved (Table 2, Figure S2), the physicochemical distance between tryptophan and arginine is high, and the variant is predicted to be pathogenic by most of the predictive tools, with a CADD score of 28.3. The p.(Met505Val) is moderately conserved (Supplementary Figure S2), the physicochemical distance between methionine and valine is small, but the variant is predicted to be pathogenic by 12 of 19 prediction tools, and the CADD score is 23. Both missense variants were classified as variants of unknown significance according to ACMG. However, since each is in trans with a known pathogenic variant, the PM3 criteria could be applied, modifying the class to probably pathogenic. A novel splice variant, c.1282-2A>G p.(?), was identified, which affects the canonical splice site (SpliceAI score 0.85, MaxEnt and SpliceSiteFinder: −100%) and is extremely rare, since gnomAD reported only one case in the non-Finnish European population (Table 2).

### 2.3. CNV Identification and Breakpoint Characterization

Only one large intragenic deletion in *TTLL5* has recently been reported [15] (Figure 2). Interestingly, we identified two additional deletions in this study and specified the breakpoints by whole genome sequencing (Supplementary Figure S3). The first one, c.585+2223_3326+5684del, p.(Pro196Glufs*47), encompassing exons 8–28, is an out-of-frame deletion, and therefore leads to a null allele. The second, c.1487+1134_3741-2607delins15, p.(Ser497_Lys1247del), removes exons 18–30, but maintains the reading frame. 

To identify a possible mechanism, the breakpoints identified in patients XZ-358338 and IM-4476 were carefully evaluated by a bioinformatic analysis (Figure S4). For c.585+2223_3326+5684del, the search for repetitive elements revealed the presence of a LINE sequence (HALIME element, 282 base pair (bp) long) at the 5’ end junction of the breakpoint, and the presence of one LTR retrotransposon at the 3’ end junction (Gypsy, 51 bp). Several regions of microhomology from one to six bp were observed. For c.1487+1134_3741-2607delins15, microhomology sequences ranging from one to six bp were also detected. A 15-bp long insertion (CCTTGGATTGTACCT) was observed at the breakpoint. The repetitive element search revealed the presence of an Alu sequence at the 5’ end junction of the breakpoint (AluJb motif, 298 bp long), and a DNA/mariner element (Tigger16b, 174 bp long) at the 3’ end junction. Oligo(G)n, inverted and direct repeats were identified at the breakpoints. Despite the identification of microhomologies, repetitive elements, and non-B DNA conformations at the breakpoints, none of these elements could clearly explain the underlying mechanisms for these complex rearrangements and no recurrence shall be expected.

## 3. Discussion

The present study reports five patients with COD/CORD/EOSRD with mutations in *TTLL5*. Six of ten alleles carried novel variants: two nonsense variants, two missense variants, one canonical splice site variant and two large deletions. All five patients were selected from three large French cohorts of IRD patients, among which 450 presented COD or CORD. *TTLL5* variants account therefore for 1.1% of COD/CORD patients, which makes *TTLL5* one of the rarest gene defect underlying cone disorders [5].

### 3.1. Phenotype Characteristics

In our series, four adult patients presented a relatively homogeneous disease course, with predominantly cone involvement (two COD and two CORD patients). Of note, three of our patients had a tritan colour defect, reported in some COD/CORD pedigrees [20,21], progressing towards complete colour blindness, as in subject IM-4476. The stage of retinal degeneration and the perifoveal cone involvement [22] were suggested as the underlying mechanism of selective tritan defect in COD/CORD. These mechanisms are not linked to variants in opsin genes and could also be non-specific [23,24]. Patient EB-163150 had a milder course of retinal degeneration at the first examination (72 y.o.) compared to the three younger patients: only cone-driven ERG responses were altered, and SWAF and SD-OCT showed a milder alteration of outer retinal structures (Figure 1b). These data are in line with previously reported phenotypes [8,13,14].

In contrast, a peculiar clinical picture of early-onset severe retinal dystrophy (EOSRD) was observed in patient IA-CIC08269. EOSRD [25], also known as early-onset retinitis pigmentosa [26,27] or severe early childhood onset retinal dystrophy (SECORD) [28], is a retinal dystrophy usually manifesting before 5–6 years of age. EOSRD is genetically and phenotypically continuum of Leber congenital amaurosis, with patients presenting a better visual function due to more preserved photoreceptors. In addition, nystagmus and eye poking are not observed, and legal blindness is reached around the age of twenty [29]. This EOSRD phenotype in patient IA-CIC08269 might be due to other variants present in *USH2A*, which must be confirmed by cosegregation analysis. Sergouniotis and colleagues reported younger patients with predominant rod photoreceptor involvement [8], but the first clinical signs were more consistent with cone dysfunction (low BCVA and light sensitivity). Another atypical picture closer to a cone dysfunction syndrome, was described in a nine-year-old boy harbouring a homozygous splice site variant in *TTLL5* [14]. The initial presentation suggested incomplete congenital stationary night blindness, but with no night blindness complaints, no peripheral vision loss and normal scotopic responses on ffERG. Interestingly, a patient with bilateral mixed hearing loss was also described by Sergouniotis et al. Patient IA-CIC08269 carried three *USH2A* variants, among which a variant of unknown significance, p.(Lys1529Ile), was previously reported in hearing loss [19]. However, no hearing defect on repeated audiometry was detected in our patient. *USH2A* gene defects lead to either syndromic or nonsyndromic IRD [30,31], and we hypothesized that the presence of both *TTLL5* and *USH2A* variants might explain the more severe phenotype of IRD, consistent with EOSRD. Similar patients carrying gene defects in more than one IRD-associated gene have already been reported [32]. 

The EOSRD phenotype raised the question of rod involvement. *TTLL5* variants have only been clearly associated with COD/CORD to date. However, some rare cases of retinitis pigmentosa (RP) presumably linked with *TTLL5* have been reported in the literature [33,34]. However, no detailed clinical description and no segregation analysis data are available. On the other hand, RP is an umbrella term, and CORD might sometimes fall under this clinical description. The involvement of *TTLL5* in RP cannot be ruled out if we consider the phenotypes associated with the connected gene *RPGR*, whose function in photoreceptor cilia requires TTLL5 glutamylation. Indeed, *Ttll5*^−/−^ and *Rpgr*^−/−^ mice share common features (early cone involvement and overall long-term disease progression) because both phenotypes are due to a nonfunctional *RPGR^ORF15^*. In humans, *RPGR^ORF15^* gene defects are observed in both CORD and RCD patients [35,36,37]. Sun et al. suggested a selection bias for CORD involvement. However, among 3300 patients with IRD tested in our laboratories, except the EOSRD patient, only patients with a COD/CORD phenotype have been found in association with *TTLL5* variants thus far. The reason for the initial rod sparing is not yet understood. Despite common molecular pathways between *TTLL5* and the *RPGR* gene, *TTLL5* variants may possibly lead to a milder disease than the *RPGR^ORF15^* variants [12]. 

### 3.2. TTLL5 Variants and Structure-Function Correlation 

All *TTLL5* variants reported to date are located throughout the sequence, but two main hot spots have emerged (Figure 2): the 5′ end of the TTLL core and a zone surrounded by the TTLL core and the cofactor interaction domain (CID), which are the two major functional domains [11,12]. Indeed, the binding of the basic domain of RPGR^ORF15^ with the CID of TTLL5 allows glutamylation of the RPGR^ORF15^ Glu-Gly repeat region by TTLL5. Most of the reported variants are frameshift, splice site or nonsense variations (10 out of 17 variants in HGMD; 59%) and can disrupt the glutamylase function. Among the novel variants reported in our study, three of them, c.211C>T, c.1920G>A and c.585+2223_3326+5684del, might lead to an absence of protein synthesis due to nonsense-mediated decay (NMD) or a shorter protein. In addition, two variants led to in-frame deletions: the large deletion c.1487+1134_3741-2607delins15 and the c.1282-2A>G splice site variant, which was predicted by all in silico tools to cause exon 16 skipping. For both variants, NMD is not expected, and a truncated and abnormal protein might be produced.

To date, only four missense variants associated with disease have been described in *TTLL5*: p.(Glu543Lys) [8], p.(Ile756Phe) [13], and the novel p.(Trp492Arg) and p.(Met505Val). These might be hypomorphic variations with a milder effect. However, the course of the disease in two homozygous carriers for the p.(Glu543Lys) variant described by Sergouniotis et al. and Bedoni et al. seems to be similar to the other patients harbouring loss-of-function mutations [8,13]. A functional study by Sun et al. showed that the p.(Glu543Lys) variant, which preserves the CID domain, severely impairs but maintains trace glutamylation activity towards RPGR^ORF15^ and tubulin. This result supports rather a strong effect of this variant. We hypothesized that polyglutamylase function is impaired not only by a complete loss of function of the protein, but also by an abnormal protein conformation altering the TTLL and CID domains, leading to reduced or lower levels of polyglutamylation activity [11,12]. 

Only one large deletion has been reported so far [15]. Large deletions represent 20% of the pathogenic *TTLL5* alleles in our study and 10.8% of the total mutational load, considering all reported patients. This suggests that large rearrangements are common in *TTLL5* and should be carefully examined. Indeed, the in-silico tool CENSOR (https://www.girinst.org/censor/) revealed the presence of a high *Alu* repeat content in the *TTLL5* intronic regions. *Alu* repeats are common short interspersed nuclear elements throughout the genome leading to recurrent rearrangements through nonallelic homologous *Alu-Alu* recombination [38]. To decipher the mechanisms underlying the development of deletions, we investigated the local genomic architecture by searching for microhomology regions, repetitive elements, and sequences forming non-B DNA conformations that could all impair the replication process. This analysis identified repetitive elements in the regions surrounding the breakpoints SINE, LINE, LTR, and DNA elements, as previously reported [39], but none of them belonged to the same class, and only one *Alu* sequence was identified, ruling out the possibility of a nonallelic homologous recombination (NAHR) that concerns rearrangements between two repetitive elements of the same family. This could explain the absence of recurrence for the reported rearrangements. Furthermore, the presence of microhomology at breakpoint junctions, ranging from one to six base pairs (bp), and of scars characterized by the insertion of several random nucleotides (15 bp in patient IM-4476) lead us to propose nonhomologous end joining (NHEJ) or replication slippage models as the main mechanisms implicated in large structural rearrangements [40]. Therefore, large deletions should be investigated by standard testing of a patient presenting COD or CORD, especially when only a single nucleotide variant is identified in *TTLL5* or in the case of a large region bearing homozygous single nucleotide variants suggestive of heterozygosity loss.

### 3.3. Genotype-Phenotype Correlations

Similar to other IRDs, phenotypic intersubject variability is not always explained by the underlying genotype. In our series, there was no evidence for a link between *TTLL5* variant type and the severity of retinal degeneration. A less severe phenotype was observed in our oldest patient harbouring a splice site defect in *trans* with a likely pathogenic missense variant (EB-163150). As discussed above, loss-of-function defects in *TTLL5* do not seem to be more severe than homozygous or compound heterozygous missense variants. For instance, in a similar study, a 53 y.o. man homozygous for p.(Glu543Lys), with adult-onset cone dystrophy and rod preservation, had the same presentation as two younger patients (38 and 45 y.o.) carrying compound heterozygous truncating mutations [8]. 

Infertility is reported in the literature but not systematically shared by the patients. Male mouse models lacking the CID or RID domains display infertility and azoospermia, suggesting that patients with truncating mutations may have a higher probability of reduced fertility [9]. In our study, two adult patients out of four had no offspring (IM-4476 and HD-2011304). Although infertility in these patients was not proven, they both carried missense or in-frame deletions, leading to an abnormal conformation of the protein. These potential discrepancies between humans and the *Ttll*^−/−^ mouse model could be explained first by redundant activities among the TTLL family members. Although different TTLL isoforms are expressed in mice in a tissue-specific manner, all isoforms are highly expressed in the testis [41]. One can speculate that, in humans, other TTLL proteins could intervene when TTLL5 is defective. Second, the balance between glutamylase and deglutamylase enzymes [10] in the manner of enzymes encoded by the *AGBL5* gene [42] can modulate polyglutamylate activity. However, further studies are required to investigate fertility in patients carrying *TTLL5* pathogenic variants.

### 3.4. Future Directions

Due to the rarity of the disorder, international collaborative studies would be necessary to define the full spectrum of *TTLL5*-related retinal disease and genotype-phenotype correlations. Middle-age onset and slow progression of retinal degeneration in most patients, combined with the availability of a murine model reproducing the human phenotype and the relatively suitable size of the gene, make *TTLL5* a good target for gene therapy approaches.

Polyglutamylases are a large and ubiquitous class of enzymes, and one can expect gene defects in other members of this family to lead to human pathologies. Complementary whole exome and whole genome sequencing studies would be helpful to assess their implication in inherited diseases, not only in retinal degeneration, but also in neurodegenerative and/or fertility disorders.

## 4. Materials and Methods

### 4.1. Subject and Clinical Examination 

Patients harbouring *TTLL5* biallelic variants were retrospectively recruited from the following French institutions: Centre de référence des maladies rares neuro-rétiniennes (REFERET), Centre Hospitalier National d’Ophtalmologie des Quinze-Vingts, Paris (IA-CIC08269), National Center for Rare Genetic Retinal Dystrophies, Hôpital Guy de Chauliac, Montpellier (IM-4476), Fondation Ophtalmologique Adolphe de Rothschild, Paris (EB-163150), Centre de référence pour les affections rares en génétique ophtalmologiques, Hôpitaux Universitaires de Strasbourg, Strasbourg (HD-2011304) and Polyclinique Jules Verne, Nantes (XZ-358338). Informed consent was obtained from all patients and/or their parents when relevant and available. The study protocol adhered to the tenets of the Declaration of Helsinki and was approved by the local ethics committee (No. 2020-A02559-30; 25 November 2020).

Clinical data were retrospectively collected from medical records. These included sex, age at the time of diagnosis and examination, personal and familial history, visual complaints, best-corrected visual acuity (BCVA) assessed by the Early Treatment Diabetic Retinopathy Study (ETDRS) chart, refractive error, slit-lamp biomicroscopy, Lanthony D-15 panel, Goldmann kinetic visual fields (VFs), full-field electroretinogram (ffERG), spectral domain optical coherence tomography (SD-OCT), fundus photography, short wavelength autofluorescence (SWAF), and near-infrared reflectance (NIR) fundus imaging. 

### 4.2. Genetic Analysis

Targeted NGS gene panel: Patients were tested by targeted NGS in three centers (gene panel details in Supplementary Table S1, and strategies are presented in Supplementary Materials). The target regions comprised the coding exons and their flanking intronic regions. The strategy used for genotyping of subject IA-CIC08269 has been described previously [5,43]. Analysis of patient HD-2011304 was performed according to methods previously described [44].

All single nucleotide variations were confirmed by Sanger sequencing of the *TTLL5* exons (OMIM 615860, NM_015072.4) (PCR-sequence conditions are available upon request). Segregation analysis was performed when family members’ DNA samples were available.

Copy number variants (CNVs): CNVs were first detected by a quantitative analysis of the data obtained from the NGS pipeline and then confirmed by quantitative PCR (qPCR) using an ABI PRISM 7900 HT instrument (Applied Biosystems, Foster City, CA, USA). Whole genome sequencing (BGI Genomics, Hong Kong) of patients IM-4476 and XZ-358338 followed by targeted analysis of *TTLL5* was also performed to delineate the breakpoints. The identification of repetitive elements and microhomology at deletion breakpoints is described in the Supplementary Materials.

Variant pathogenicity assessment: Variant pathogenicity was assessed through our in-house pipeline embedding commercially available bioinformatics software and using data available from public variant databases in accordance with the Guidelines of the American College of Medical Genetics and Genomics [18]. More details are provided in the Supplementary Materials.

## Figures and Tables

**Table 1:** Clinical characteristics of patients.

Patient, Sex	Symptoms	BCVA at First Visit	Refraction	Anterior Segment	Fundus	Colour Vision	Visual Field	ffERG	SWAF	SD-OCT
IA-CIC08269, F	Night vision difficulties since 6 y.o. Bilateral decreased visual acuity	RE: 20/50 LE: 20/50 7 y.o.	+1.0(−1.5)10° +1.0(−1.5)175°	Normal	Normal optic discs Mild vascular narrowing Dark fovea, yellowish deposit with indistinct borders surrounding the fovea Whitish spots of chorio-retinal atrophy and coarse pigmentary migration in peripheral retina	Tritan defect	Kinetic perimetry: V4e: 145° horizontal and 120° vertical III1e: constricted to 5° I1e: not perceived OU	Undetectable scotopic- and photopic responses	Very narrow ring of increased autofluorescence	Outer retinal layers (EZ, ONL) disappearance outside the fovea
XZ-358338, M	Progressive visual loss since teens, Photophobia from age 36 y.o.	RE: 20/50 LE: 20/40 36 y.o.	−12.5(−2.0)0° −12.0(−3.0)160°	Phakic IOL implanted at 36 y.o.	Normal discs Peripapillary atrophy (conus myopicus) Mild arteriolar narrowing Dark round atrophic lesion of the macula with indistinct borders Normal peripheral retina	Tritan defect	Static perimetry: Absolute central scotoma of 5° surrounded by a ring of reduced sensitivity OU	Generalized cone-rod dysfunction (scotopic responses 70% of normal amplitude and photopic responses 25% of normal amplitude)	Salt and pepper round macular lesion with hyperautofluorescent edges Normal autofluorescence in the peripheral retina	Dome-shaped macula Outer retinal layers (EZ, ONL) disappearance in the macula; normal aspect outside the macular lesion
IM-4476, M	Progressive visual acuity loss since childhood, Photophobia and rapid visual acuity loss since 42 y.o.	RE:20/100 LE: 20/50 46 y.o.	−6.25(−3.0)170° −6.0(−3.50)35°	Normal	Normal discs Chorioretinal thinning between the macula and optic disc Yellowish atrophic round macular lesion Normal peripheral retina	Severe dyschromatopsia	V4e: 150° horizontal and 120° vertical II4e: 90° horizontal and 60° vertical I4e not perceived OU	Severe generalized cone system dysfunction with normal rod system function	Round foveal hypoautofluorescent lesion with irregular hyperautofluorescent edges	Foveal disappearance of outer retinal layers (EZ, ONL)
HD-2011304, M	Night vision disturbances since his forties Photophobia and rapid visual acuity loss since 45 y.o.	RE:20/63 LE:20/50 48 y.o.	-6.75(−3.50)80° −9.50(−3.50)110°	Normal	Normal disc Peripapillary atrophy (conus myopicus) Mild arteriolar narrowing Dark foveal spot surrounded by ovoid zone of macular discoloration Normal peripheral retina	Tritan defect	V4e: 100° horizontal and 90° vertical V4e central scotoma of 15°	Generalized cone-rod dysfunction (scotopic responses 50% of normal amplitude, photopic responses 30% of normal amplitude)	Foveal hyperautofluorescent spot surrounded by a round hypoautofluorescent area with hyperautofluorescent edges Macular lesion is continuous with the peripapillary atrophy	Perifoveal disappearance of outer retinal layers (EZ, ONL) Foveal sparing of EZ
EB-163150, M	Progressive visual acuity loss from teens Photophobia since childhood, became disabling from thirties	RE: 20/80 LE: 20/80 70 y.o.	−6.0(−1.0)40° −7.0(−0.75)165°	Cortico-nuclear and posterior subcapsular cataract OU	Normal disc Peripapillary atrophy (conus myopicus) Mild arteriolar narrowing Round macular discoloration	Tritan defect	V4e: 140° horizontal and vertical V1e: central scotoma of 15° Humphrey 10-2: sparing of fixation point, absolute annular scotoma at 5° of eccentricity surrounded by a relative deficit	Severe cone system dysfunction with only residual photopic responses while scotopic responses were normal	Perifoveal hyperautofluorescent ring with indistinct edges	Perifoveal disappearance of outer retinal layers (EZ, ONL) Foveal sparing of EZ

BCVA—best corrected visual acuity, EZ—ellipsoid zone, ONL—outer nuclear layer, OU—both eyes, NA—Not applicable, SWAF—short wave fundus autofluorescence, SD-OCT—spectral domain optical coherence tomography, ffERG—full-field electroretinography.

**Table 2:** In silico variant analysis.

Patient ID	Status	Genomic Position	DNA Variant	Protein Variant	Variant Type	GnomAD AF (NFE)	In Silico Prediction		ACMG Rules	ACMG Classification	Reference
IA-CIC08269	Homozygous	76232616	c.1920G>A	p.(Trp640*)	Nonsense	0	CADD	36.0	PVS1 Very strong	Pathogenic	This study
									PM2 Moderate		
									PP3 Supporting		
									PP4 Supporting		
									PP5 Supporting		
XZ-358338	Compound Heterozygous	76147917	c.211C>T	p.(Arg71*)	Nonsense	0.00002	CADD	35.0	PVS1 Very strong	Pathogenic	Zampaglione 2020
									PM2 Moderate		
									PM3 Moderate		
									PP3 Supporting		
									PP4 Supporting		
									PP5 Supporting		
		76167836_ 76292188	c.585+2223_3326+5684del	p.(Pro196Glufs*47)	Exons 8-28 deletion	0			PVS1 Very strong	Pathogenic	This study
									PM2 Moderate		
									PM3 Moderate		
									PP3 Supporting		
									PP4 Supporting		
									PP5 Supporting		
IM-4476	Compound Heterozygous	76213058_ 76365878	c.1487+1134_3741- 2607delins15	p.(Ser497_Lys1247del)	Exons 18-30 deletion	0			PVS1 Very strong	Pathogenic	This study
									PM2 Moderate		
									PP3 Supporting		
									PP4 Supporting		
									PP5 Supporting		
		76231034	c.1627G>A	p.(Glu543Lys)	Missense	0.0003	Conservation (Grantham)	56 (0–215)	PS3 Strong	Pathogenic	Sergouniotis 2014
							CADD	27.6	PM2 Moderate		
							SIFT	Deleterious (score: 0.02)	PP3 Supporting		
							Polyphen2	Probably Damaging (1.000)	PP4 Supporting		
									PP5 Supporting		
HD-2011304	Compound Heterozygous	76211911	c.1474T>A	p.(Trp492Arg)	Missense	0	Conservation (Grantham)	101 (0–215)	PM2 Moderate	Likely Pathogenic *	This study
							CADD	28.3	PM3 Moderate		
							SIFT	Deleterious (score: 0)	PP3 Supporting		
							Polyphen2	Probably Damaging (0.995)	PP4 Supporting		
		76231034	c.1627G>A	p.(Glu543Lys)	Missense	0.0003	Conservation (Grantham)	56 (0–215)	PS3 Strong	Pathogenic	Sergouniotis 2014
							CADD	27.6	PM2 Moderate		
							SIFT	Deleterious (score: 0.02)	PP3 Supporting		
							Polyphen2	Probably Damaging (1.000)	PP4 Supporting		
									PP5 Supporting		
EB-163150	Compound Heterozygous	76211436	c.1282-2A>G	p.(?)	Splicing	0.000009	CADD	34.0	PVS1 Very strong	Pathogenic	This study
							SpliceSiteFinder	−100%	PM2 Moderate		
							MaxEnt	−100%	PP3 Supporting		
							SpliceAI	0.85	PP4 Supporting		
		76219261	c.1513A>G	p.(Met505Val)	Missense	0.00005	Conservation (Grantham)	21 (0–215)	PM2 Moderate	Likely Pathogenic *	This study
							CADD	23.0	PM3 Moderate		
							SIFT	Tolerated (score: 0.2)	PP3 Supporting		
							Polyphen2	Possibly Damaging (0.786)	PP4 Supporting		

* Stand Alone VUS becoming Likely Pathogenic with a pathogenic variant in *trans.*

## Data Availability

All data are contained within the article or Supplementary Material.

## Supplementary Materials

The following are available online at https://www.mdpi.com/article/10.3390/ijms22126410/s1, Figure S1: Pedigrees of families with TTLL5 variants, Figure S2: Conservation analysis of the novel missense variants on TTLL5, Figure S3: Large multiexons deletions depicted by IGV screenshots, Figure S4: Multiple sequence alignment and schematic overview of breakpoint analysis, Table S1: List of genes tested.

## References

[1] Hamel C.P. (2007). Cone Rod Dystrophies. Orphanet J. Rare Dis..

[2] Michaelides M., Hardcastle A.J., Hunt D.M., Moore A.T. (2006). Progressive Cone and Cone-Rod Dystrophies: Phenotypes and Underlying Molecular Genetic Basis. Surv. Ophthalmol..

[3] Thiadens A.A.H.J., Phan T.M.L., Zekveld-Vroon R.C., Leroy B.P., van den Born L.I., Hoyng C.B., Klaver C.C.W., Roosing S., Pott J.-W.R., Writing Committee for the Cone Disorders Study Group Consortium (2012). Clinical Course, Genetic Etiology, and Visual Outcome in Cone and Cone-Rod Dystrophy. Ophthalmology.

[4] Michaelides M., Hunt D.M., Moore A.T. (2004). The Cone Dysfunction Syndromes. Br. J. Ophthalmol..

[5] Boulanger-Scemama E., El Shamieh S., Démontant V., Condroyer C., Antonio A., Michiels C., Boyard F., Saraiva J.-P., Letexier M., Souied E. (2015). Next-Generation Sequencing Applied to a Large French Cone and Cone-Rod Dystrophy Cohort: Mutation Spectrum and New Genotype-Phenotype Correlation. Orphanet J. Rare Dis..

[6] Gill J.S., Georgiou M., Kalitzeos A., Moore A.T., Michaelides M. (2019). Progressive Cone and Cone-Rod Dystrophies: Clinical Features, Molecular Genetics and Prospects for Therapy. Br. J. Ophthalmol..

[7] Tsang S.H., Sharma T. (2018). Progressive Cone Dystrophy and Cone-Rod Dystrophy (XL, AD, and AR). Adv. Exp. Med. Biol..

[8] Sergouniotis P.I., Chakarova C., Murphy C., Becker M., Lenassi E., Arno G., Lek M., MacArthur D.G., Bhattacharya S.S., UCL-Exomes Consortium (2014). Biallelic Variants in TTLL5, Encoding a Tubulin Glutamylase, Cause Retinal Dystrophy. Am. J. Hum. Genet..

[9] Lee G.-S., He Y., Dougherty E.J., Jimenez-Movilla M., Avella M., Grullon S., Sharlin D.S., Guo C., Blackford J.A., Awasthi S. (2013). Disruption of Ttll5/Stamp Gene (Tubulin Tyrosine Ligase-like Protein 5/SRC-1 and TIF2-Associated Modulatory Protein Gene) in Male Mice Causes Sperm Malformation and Infertility. J. Biol. Chem..

[10] van Dijk J., Miro J., Strub J.-M., Lacroix B., van Dorsselaer A., Edde B., Janke C. (2008). Polyglutamylation Is a Post-Translational Modification with a Broad Range of Substrates. J. Biol. Chem..

[11] Mahalingan K.K., Keith Keenan E., Strickland M., Li Y., Liu Y., Ball H.L., Tanner M.E., Tjandra N., Roll-Mecak A. (2020). Structural Basis for Polyglutamate Chain Initiation and Elongation by TTLL Family Enzymes. Nat. Struct. Mol. Biol..

[12] Sun X., Park J.H., Gumerson J., Wu Z., Swaroop A., Qian H., Roll-Mecak A., Li T. (2016). Loss of RPGR Glutamylation Underlies the Pathogenic Mechanism of Retinal Dystrophy Caused by TTLL5 Mutations. Proc. Natl. Acad. Sci. USA.

[13] Bedoni N., Haer-Wigman L., Vaclavik V., Tran V.H., Farinelli P., Balzano S., Royer-Bertrand B., El-Asrag M.E., Bonny O., Ikonomidis C. (2016). Mutations in the Polyglutamylase Gene *TTLL5*, Expressed in Photoreceptor Cells and Spermatozoa, Are Associated with Cone-Rod Degeneration and Reduced Male Fertility. Hum. Mol. Genet..

[14] Dias M.d.S., Hamel C.P., Meunier I., Varin J., Blanchard S., Boyard F., Sahel J.-A., Zeitz C. (2017). Novel Splice-Site Mutation in TTLL5 Causes Cone Dystrophy in a Consanguineous Family. Mol. Vis..

[15] Méjécase C., Kozak I., Moosajee M. (2020). The Genetic Landscape of Inherited Eye Disorders in 74 Consecutive Families from the United Arab Emirates. Am. J. Med. Genet. C Semin. Med. Genet..

[16] Patel N., Alkuraya H., Alzahrani S.S., Nowailaty S.R., Seidahmed M.Z., Alhemidan A., Ben-Omran T., Ghazi N.G., Al-Aqeel A., Al-Owain M. (2018). Mutations in Known Disease Genes Account for the Majority of Autosomal Recessive Retinal Dystrophies. Clin. Genet..

[17] Zampaglione E., Kinde B., Place E.M., Navarro-Gomez D., Maher M., Jamshidi F., Nassiri S., Mazzone J.A., Finn C., Schlegel D. (2020). Copy-Number Variation Contributes 9% of Pathogenicity in the Inherited Retinal Degenerations. Genet. Med. Off. J. Am. Coll. Med. Genet..

[18] Richards S., Aziz N., Bale S., Bick D., Das S., Gastier-Foster J., Grody W.W., Hegde M., Lyon E., Spector E. (2015). Standards and Guidelines for the Interpretation of Sequence Variants: A Joint Consensus Recommendation of the American College of Medical Genetics and Genomics and the Association for Molecular Pathology. Genet. Med. Off. J. Am. Coll. Med. Genet..

[19] Sloan-Heggen C.M., Bierer A.O., Shearer A.E., Kolbe D.L., Nishimura C.J., Frees K.L., Ephraim S.S., Shibata S.B., Booth K.T., Campbell C.A. (2016). Comprehensive Genetic Testing in the Clinical Evaluation of 1119 Patients with Hearing Loss. Hum. Genet..

[20] van Schooneveld M.J., Went L.N., Oosterhuis J.A. (1991). Dominant Cone Dystrophy Starting with Blue Cone Involvement. Br. J. Ophthalmol..

[21] Kohl S., Llavona P., Sauer A., Reuter P., Weisschuh N., Kempf M., Dehmelt F.A., Arrenberg A.B., Sliesoraityte I., Zrenner E. (2021). A Duplication on Chromosome 16q12 Affecting the IRXB Gene Cluster Is Associated with Autosomal Dominant Cone Dystrophy with Early Tritanopic Color Vision Defect. Hum. Mol. Genet..

[22] Pinckers A. (1992). Dominant Cone Dystrophy Starting with Blue Cone Involvement. Br. J. Ophthalmol..

[23] Zrenner E., Nowicki J., Adamczyk R. (1986). Cone Function and Cone Interaction in Hereditary Degenerations of the Central Retina. Doc. Ophthalmol..

[24] Simunovic M.P. (2016). Acquired Color Vision Deficiency. Surv. Ophthalmol..

[25] Kumaran N., Moore A.T., Weleber R.G., Michaelides M. (2017). Leber Congenital Amaurosis/Early-Onset Severe Retinal Dystrophy: Clinical Features, Molecular Genetics and Therapeutic Interventions. Br. J. Ophthalmol..

[26] Foxman S.G., Heckenlively J.R., Bateman J.B., Wirtschafter J.D. (1985). Classification of Congenital and Early Onset Retinitis Pigmentosa. Arch. Ophthalmol. Chic. Ill. 1960.

[27] Lorenz B., Gyürüs P., Preising M., Bremser D., Gu S., Andrassi M., Gerth C., Gal A. (2000). Early-Onset Severe Rod-Cone Dystrophy in Young Children with RPE65 Mutations. Invest. Ophthalmol. Vis. Sci..

[28] Weleber R.G., Michaelides M., Trzupek K.M., Stover N.B., Stone E.M. (2011). The Phenotype of Severe Early Childhood Onset Retinal Dystrophy (SECORD) from Mutation of RPE65 and Differentiation from Leber Congenital Amaurosis. Invest. Ophthalmol. Vis. Sci..

[29] Kumaran N., Pennesi M.E., Yang P., Trzupek K.M., Schlechter C., Moore A.T., Weleber R.G., Michaelides M., Adam M.P., Ardinger H.H., Pagon R.A., Wallace S.E., Bean L.J., Mirzaa G., Amemiya A. (1993). Leber Congenital Amaurosis / Early-Onset Severe Retinal Dystrophy Overview. GeneReviews^®^.

[30] McGee T.L., Seyedahmadi B.J., Sweeney M.O., Dryja T.P., Berson E.L. (2010). Novel Mutations in the Long Isoform of the USH2A Gene in Patients with Usher Syndrome Type II or Non-Syndromic Retinitis Pigmentosa. J. Med. Genet..

[31] Seyedahmadi B.J., Rivolta C., Keene J.A., Berson E.L., Dryja T.P. (2004). Comprehensive Screening of the USH2A Gene in Usher Syndrome Type II and Non-Syndromic Recessive Retinitis Pigmentosa. Exp. Eye Res..

[32] Méjécase C., Hummel A., Mohand-Saïd S., Andrieu C., El Shamieh S., Antonio A., Condroyer C., Boyard F., Foussard M., Blanchard S. (2019). Whole Exome Sequencing Resolves Complex Phenotype and Identifies *CC2D2A* Mutations Underlying Non-Syndromic Rod-Cone Dystrophy. Clin. Genet..

[33] Gao F.-J., Li J.-K., Chen H., Hu F.-Y., Zhang S.-H., Qi Y.-H., Xu P., Wang D.-D., Wang L.-S., Chang Q. (2019). Genetic and Clinical Findings in a Large Cohort of Chinese Patients with Suspected Retinitis Pigmentosa. Ophthalmology.

[34] Sharon D., Ben-Yosef T., Goldenberg-Cohen N., Pras E., Gradstein L., Soudry S., Mezer E., Zur D., Abbasi A.H., Zeitz C. (2020). A Nationwide Genetic Analysis of Inherited Retinal Diseases in Israel as Assessed by the Israeli Inherited Retinal Disease Consortium (IIRDC). Hum. Mutat..

[35] Di Iorio V., Karali M., Melillo P., Testa F., Brunetti-Pierri R., Musacchia F., Condroyer C., Neidhardt J., Audo I., Zeitz C. (2020). Spectrum of Disease Severity in Patients With X-Linked Retinitis Pigmentosa Due to RPGR Mutations. Invest. Ophthalmol. Vis. Sci..

[36] Hadalin V., Šuštar M., Volk M., Maver A., Sajovic J., Jarc-Vidmar M., Peterlin B., Hawlina M., Fakin A. (2021). Cone Dystrophy Associated with a Novel Variant in the Terminal Codon of the RPGR-ORF15. Genes.

[37] Nguyen X.-T.-A., Talib M., van Schooneveld M.J., Brinks J., Ten Brink J., Florijn R.J., Wijnholds J., Verdijk R.M., Bergen A.A., Boon C.J.F. (2020). RPGR-Associated Dystrophies: Clinical, Genetic, and Histopathological Features. Int. J. Mol. Sci..

[38] Vissers L.E.L.M., Bhatt S.S., Janssen I.M., Xia Z., Lalani S.R., Pfundt R., Derwinska K., de Vries B.B.A., Gilissen C., Hoischen A. (2009). Rare Pathogenic Microdeletions and Tandem Duplications Are Microhomology-Mediated and Stimulated by Local Genomic Architecture. Hum. Mol. Genet..

[39] Van Schil K., Naessens S., Van de Sompele S., Carron M., Aslanidis A., Van Cauwenbergh C., Mayer A.K., Van Heetvelde M., Bauwens M., Verdin H. (2019). Correction: Mapping the Genomic Landscape of Inherited Retinal Disease Genes Prioritizes Genes Prone to Coding and Noncoding Copy-Number Variations. Genet. Med. Off. J. Am. Coll. Med. Genet..

[40] Lieber M.R. (2010). The Mechanism of Double-Strand DNA Break Repair by the Nonhomologous DNA End-Joining Pathway. Annu. Rev. Biochem..

[41] van Dijk J., Rogowski K., Miro J., Lacroix B., Eddé B., Janke C. (2007). A Targeted Multienzyme Mechanism for Selective Microtubule Polyglutamylation. Mol. Cell.

[42] Astuti G.D.N., Arno G., Hull S., Pierrache L., Venselaar H., Carss K., Raymond F.L., Collin R.W.J., Faradz S.M.H., van den Born L.I. (2016). Mutations in AGBL5, Encoding α-Tubulin Deglutamylase, Are Associated With Autosomal Recessive Retinitis Pigmentosa. Invest. Ophthalmol. Vis. Sci..

[43] Audo I., Bujakowska K.M., Léveillard T., Mohand-Saïd S., Lancelot M.-E., Germain A., Antonio A., Michiels C., Saraiva J.-P., Letexier M. (2012). Development and Application of a Next-Generation-Sequencing (NGS) Approach to Detect Known and Novel Gene Defects Underlying Retinal Diseases. Orphanet J. Rare Dis..

[44] Geoffroy V., Stoetzel C., Scheidecker S., Schaefer E., Perrault I., Bär S., Kröll A., Delbarre M., Antin M., Leuvrey A.-S. (2018). Whole-Genome Sequencing in Patients with Ciliopathies Uncovers a Novel Recurrent Tandem Duplication in IFT140. Hum. Mutat..

