# Supplementary material for: Novel TTLL5 Variants Associated with Cone-Rod Dystrophy and Early-Onset Severe Retinal Dystrophy
Source: Int J Mol Sci. 2021 Jun 15;22(12):6410. doi: 10.3390/ijms22126410 (PMC8232641; doi:10.3390/ijms22126410)
Supplement: Supplementary file 1 [file ijms-22-06410-s001.zip › ijms-1247265-supplementary file_modified.pdf]

Novel *TTLL5* Variants Associated with Cone-Rod Dystrophy and Early-Onset Severe Retinal Dystrophy

Supplementary Materials

Targeted NGS gene panel

Different gene panels were used, containing 156 IRD genes (patients XZ-358338 and EB-163150), 208 genes (patient IM-4476), 266 genes (patient HD-2011304), and 124 genes (patient IA-CIC08269) (see the panels list in Table S1). The target regions were comprised of the coding exons and their flanking intronic regions. Capture oligonucleotide probes were designed using the HaloPlex Target Enrichment System (Agilent Technologies Inc., Santa Clara, CA, USA) according to the manufacturer's protocol. DNA libraries were sequenced on a NextSeq500 sequencer (Illumina Inc., San Diego, CA, USA). The generated sequences were analysed using an in-house developed pipeline, compiling the data obtained from Seqnext (JSI Medical System, Ettenheim, Germany) and MiSeq reporter (Illumina) software. The Institut de la Vision strategy was previously described [1,2].

Targeted exome sequencing of 266 genes related to retinitis pigmentosa was performed. Exons of DNA samples were captured by an in-solution enrichment methodology (Agilent QXT SureSelect custom panel) and sequenced with an Illumina NextSeq 550 instrument (paired-end sequencing 2x150 bases, 48 libraries per lane). SNVs and indels were called with the Genome Analysis Toolkit v.3.4.46 with the help of our in-house pipeline (STARK) and following the GATK best practice. Annotation and ranking of SNVs and indels were performed by VaRank [3]. CNVs were called using the CANOES program [4] and annotated with AnnotSV [5].

Variant validation

Sanger sequencing of *TTLL5* exons and exon-intron boundaries (OMIM 615860, NM_015072.4) was performed to confirm the presence of the variations obtained by NGS (PCR and sequencing conditions available under request). PCR products were purified on a P10 gel (Bio-Rad, Hercules, USA) and bidirectionally sequenced using the BigDye® Terminator v3.1 Cycle Sequencing Kit on a 3730 DNA Analyzer (Applied Biosystems, Carlsbad, USA).

CNV detection

The detection of CNVs was performed by a quantitative analysis of the data obtained from the bam files. For each patient and for each target (i.e., exon), we calculated the ratio as follows: the depth of the reads for the target divided by the depth of the reads for all targets, and this ratio was divided by the

full mean coverage for all control samples analysed on the same NGS run. A ratio value of 1 indicated that the copy number was identical to that of the control samples, and a ratio value of 0.5 revealed only one copy of the allele and a heterozygous deletion. CNV was confirmed by quantitative PCR (qPCR) using an ABI PRISM 7900 HT instrument (Applied Biosystems) in triplicate for each sample, according to the manufacturer's protocol.

Identification of repetitive elements and microhomology at deletion breakpoints

To decipher the mechanisms underlying large deletions, repetitive elements were searched by submitting the 150-bp segments flanking each breakpoint to the RepeatMasker (http://www.repeatmasker.org) and Censor (https://www.girinst.org/censor/) [6] online software tools. The presence of microhomology at the breakpoints was assessed by multiple sequence alignment between the junction fragment and the proximal and distal breakpoint regions using Clustal Omega (https://www.ebi.ac.uk/Tools/msa/clustalo/) [7]. Possible involvement of non-B DNA conformation elements in deletion formation was assessed with two different online software tools: Quadruplex forming G-Rich Sequences (QGRS) Mapper (http://bioinformatics.ramapo.edu/QGRS/analyze.php) [8] to identify (Oligo)n tracts forming tetraplex structures and the non-B DNA motif search tool (nBMST) (https://nonb-abcc.ncifcrf.gov/apps/nBMST/default) [9] to provide the location for direct repeats and slipped motifs, G quadruplex forming repeats, inverted repeats and cruciform motifs, mirror repeats and triplex motifs, Z-DNA motifs, and short tandem repeats.

Variant pathogenicity assessment

The pathogenicity of missense variants was assessed by the use of in-silico prediction tools using Alamut Visual software version 2.11 (Biosoftware, 2018; Interactive Biosoftware, Rouen, France; www.interactivebiosoftware) (i.e., Mutation Taster [http://www.mutationtaster.org/ [10]], Sorting Intolerant FromTolerant SIFT (https://sift.bii.a-star.edu.sg/ [11]), Polymorphism Phenotyping v2 Polyphen-2 (http://genetics.bwh.harvard.edu/pph2/ [12]), and the Varsome program [13], which queried multiple prediction tools. The Combined Annotation Dependent Depletion (CADD) score was also used [14]. The pathogenicity of splice site variants was determined by using five different algorithms, including SpliceSiteFinder-like (SSFL), MaxEntScan, NNSPLICE, GeneSplicer, and Human Splicing Finder (HSF), using Alamut Visual software version 2.11 [15–19] and SpliceAI [20]. Grantham [21] and PhyloP [22] scores were also assessed to predict the physiochemical effect of nucleotide changes on the TTLL5 protein and conservation of the nucleotide positions, respectively. Guidelines from the American College of Medical Genetics and Genomics were used for variant classification [23].

1. Boulanger-Scemama, E.; El Shamieh, S.; Démontant, V.; Condroyer, C.; Antonio, A.; Michiels, C.; Boyard, F.; Saraiva, J.-P.; Letexier, M.; Souied, E.; et al. Next-Generation Sequencing Applied to a Large French Cone and Cone-Rod Dystrophy Cohort: Mutation Spectrum and New Genotype-Phenotype Correlation. *Orphanet J. Rare Dis.* **2015**, *10*, 85, doi:10.1186/s13023-015-0300-3.
2. Audo, I.; Bujakowska, K.M.; Léveillard, T.; Mohand-Saïd, S.; Lancelot, M.-E.; Germain, A.; Antonio, A.; Michiels, C.; Saraiva, J.-P.; Letexier, M.; et al. Development and Application of a Next-Generation-Sequencing (NGS) Approach to Detect Known and Novel Gene Defects Underlying Retinal Diseases. *Orphanet J. Rare Dis.* **2012**, *7*, 8, doi:10.1186/1750-1172-7-8.
3. Geoffroy, V.; Pizot, C.; Redin, C.; Piton, A.; Vasli, N.; Stoetzel, C.; Blavier, A.; Laporte, J.; Muller, J. VaRank: A Simple and Powerful Tool for Ranking Genetic Variants. *PeerJ* **2015**, *3*, e796, doi:10.7717/peerj.796.
4. Backenroth, D.; Homsy, J.; Murillo, L.R.; Glessner, J.; Lin, E.; Brueckner, M.; Lifton, R.; Goldmuntz, E.; Chung, W.K.; Shen, Y. CANOES: Detecting Rare Copy Number Variants from Whole Exome Sequencing Data. *Nucleic Acids Res.* **2014**, *42*, e97, doi:10.1093/nar/gku345.
5. Geoffroy, V.; Herenger, Y.; Kress, A.; Stoetzel, C.; Piton, A.; Dollfus, H.; Muller, J. AnnotSV: An Integrated Tool for Structural Variations Annotation. *Bioinforma. Oxf. Engl.* **2018**, *34*, 3572–3574, doi:10.1093/bioinformatics/bty304.
6. Kohany, O.; Gentles, A.J.; Hankus, L.; Jurka, J. Annotation, Submission and Screening of Repetitive Elements in Repbase: RepbaseSubmitter and Censor. *BMC Bioinformatics* **2006**, *7*, 474, doi:10.1186/1471-2105-7-474.
7. Sievers, F.; Wilm, A.; Dineen, D.; Gibson, T.J.; Karplus, K.; Li, W.; Lopez, R.; McWilliam, H.; Remmert, M.; Söding, J.; et al. Fast, Scalable Generation of High-Quality Protein Multiple Sequence Alignments Using Clustal Omega. *Mol. Syst. Biol.* **2011**, *7*, 539, doi:10.1038/msb.2011.75.
8. Kikin, O.; D'Antonio, L.; Bagga, P.S. QGRS Mapper: A Web-Based Server for Predicting G-Quadruplexes in Nucleotide Sequences. *Nucleic Acids Res.* **2006**, *34*, W676–W682, doi:10.1093/nar/gkl253.
9. Cer, R.Z.; Donohue, D.E.; Mudunuri, U.S.; Temiz, N.A.; Loss, M.A.; Starner, N.J.; Halusa, G.N.; Volfovsky, N.; Yi, M.; Luke, B.T.; et al. Non-B DB v2.0: A Database of Predicted Non-B DNA-Forming Motifs and Its Associated Tools. *Nucleic Acids Res.* **2013**, *41*, D94–D100, doi:10.1093/nar/gks955.
10. Schwarz, J.M.; Cooper, D.N.; Schuelke, M.; Seelow, D. MutationTaster2: Mutation Prediction for the Deep-Sequencing Age. *Nat. Methods* **2014**, *11*, 361–362, doi:10.1038/nmeth.2890.
11. Sim, N.-L.; Kumar, P.; Hu, J.; Henikoff, S.; Schneider, G.; Ng, P.C. SIFT Web Server: Predicting Effects of Amino Acid Substitutions on Proteins. *Nucleic Acids Res.* **2012**, *40*, W452-457, doi:10.1093/nar/gks539.
12. Adzhubei, I.A.; Schmidt, S.; Peshkin, L.; Ramensky, V.E.; Gerasimova, A.; Bork, P.; Kondrashov, A.S.; Sunyaev, S.R. A Method and Server for Predicting Damaging Missense Mutations. *Nat. Methods* **2010**, *7*, 248–249, doi:10.1038/nmeth0410-248.
13. Kopanos, C.; Tsiolkas, V.; Kouris, A.; Chapple, C.E.; Albarca Aguilera, M.; Meyer, R.; Massouras, A. VarSome: The Human Genomic Variant Search Engine. *Bioinforma. Oxf. Engl.* **2019**, *35*, 1978–1980, doi:10.1093/bioinformatics/bty897.
14. Kircher, M.; Witten, D.M.; Jain, P.; O'Roak, B.J.; Cooper, G.M.; Shendure, J. A General Framework for Estimating the Relative Pathogenicity of Human Genetic Variants. *Nat. Genet.* **2014**, *46*, 310–315, doi:10.1038/ng.2892.
15. Cartegni, L.; Wang, J.; Zhu, Z.; Zhang, M.Q.; Krainer, A.R. ESEfinder: A Web Resource to Identify Exonic Splicing Enhancers. *Nucleic Acids Res.* **2003**, *31*, 3568–3571, doi:10.1093/nar/gkg616.
16. Desmet, F.-O.; Hamroun, D.; Lalande, M.; Collod-Béroud, G.; Claustres, M.; Béroud, C. Human Splicing Finder: An Online Bioinformatics Tool to Predict Splicing Signals. *Nucleic Acids Res.* **2009**, *37*, e67, doi:10.1093/nar/gkp215.
17. Pertea, M.; Lin, X.; Salzberg, S.L. GeneSplicer: A New Computational Method for Splice Site Prediction. *Nucleic Acids Res.* **2001**, *29*, 1185–1190, doi:10.1093/nar/29.5.1185.

18. Reese, M.G.; Eeckman, F.H.; Kulp, D.; Haussler, D. Improved Splice Site Detection in Genie. *J. Comput. Biol. J. Comput. Mol. Cell Biol.* **1997**, *4*, 311–323, doi:10.1089/cmb.1997.4.311.
19. Yeo, G.; Burge, C.B. Maximum Entropy Modeling of Short Sequence Motifs with Applications to RNA Splicing Signals. *J. Comput. Biol. J. Comput. Mol. Cell Biol.* **2004**, *11*, 377–394, doi:10.1089/1066527041410418.
20. Jaganathan, K.; Kyriazopoulou Panagiotopoulou, S.; McRae, J.F.; Darbandi, S.F.; Knowles, D.; Li, Y.I.; Kosmicki, J.A.; Arbelaez, J.; Cui, W.; Schwartz, G.B.; et al. Predicting Splicing from Primary Sequence with Deep Learning. *Cell* **2019**, *176*, 535-548.e24, doi:10.1016/j.cell.2018.12.015.
21. Grantham, R. Amino Acid Difference Formula to Help Explain Protein Evolution. *Science* **1974**, *185*, 862–864, doi:10.1126/science.185.4154.862.
22. Pollard, K.S.; Hubisz, M.J.; Rosenbloom, K.R.; Siepel, A. Detection of Nonneutral Substitution Rates on Mammalian Phylogenies. *Genome Res.* **2010**, *20*, 110–121, doi:10.1101/gr.097857.109.
23. Richards, S.; Aziz, N.; Bale, S.; Bick, D.; Das, S.; Gastier-Foster, J.; Grody, W.W.; Hegde, M.; Lyon, E.; Spector, E.; et al. Standards and Guidelines for the Interpretation of Sequence Variants: A Joint Consensus Recommendation of the American College of Medical Genetics and Genomics and the Association for Molecular Pathology. *Genet. Med. Off. J. Am. Coll. Med. Genet.* **2015**, *17*, 405–424, doi:10.1038/gim.2015.30.
