# Supplementary material for: Novel TTLL5 Variants Associated with Cone-Rod Dystrophy and Early-Onset Severe Retinal Dystrophy
Source: Int J Mol Sci. 2021 Jun 15;22(12):6410. doi: 10.3390/ijms22126410 (PMC8232641; doi:10.3390/ijms22126410)

Supplemental Figure S1: Pedigrees of families with *TTLL5* variants. Filled symbols represent affected individuals, and unfilled symbols represent unaffected individual; squares: males; circles: females.

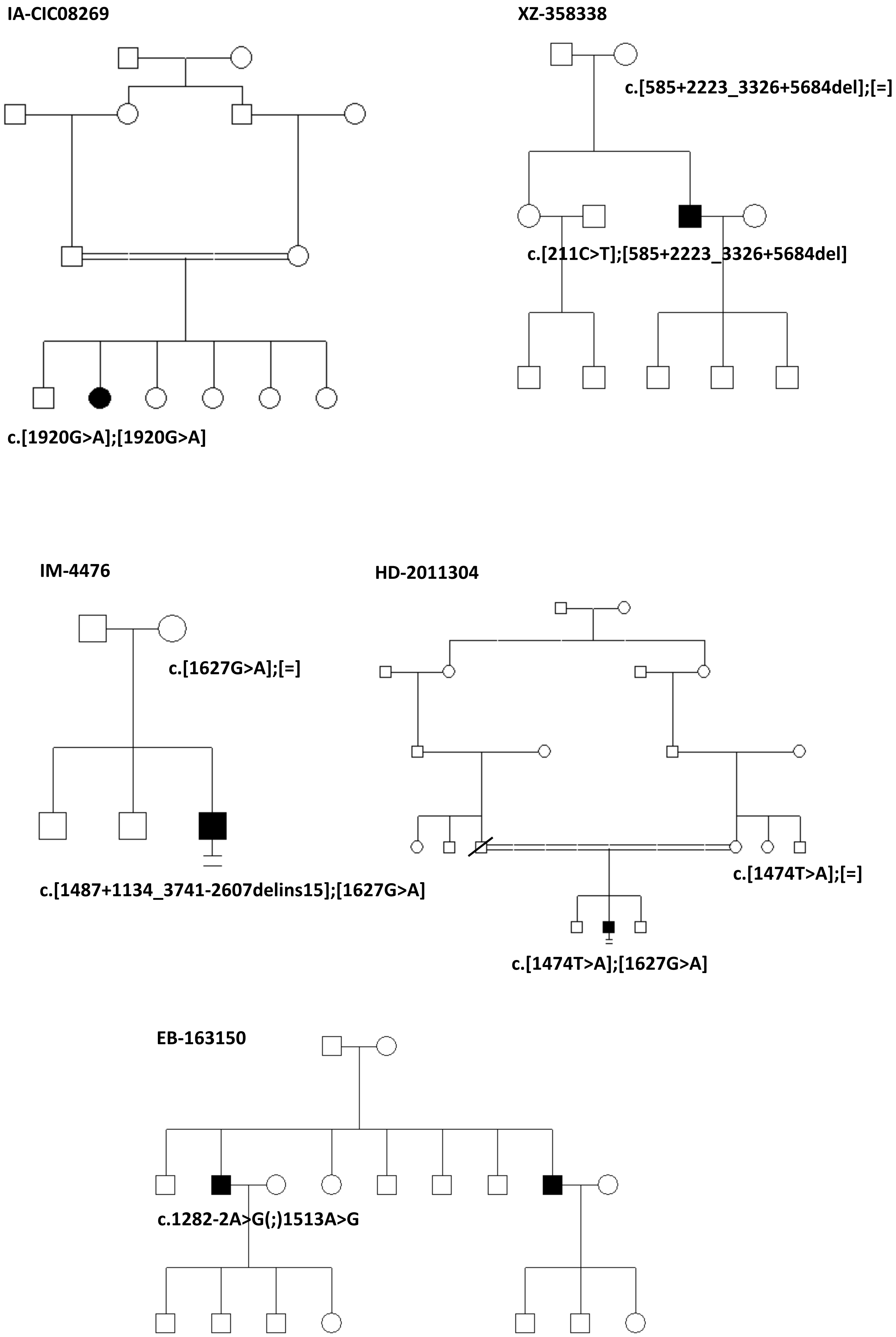

Supplement: Supplementary file 1 [file ijms-22-06410-s001.zip › Supplemental Figure S1_Pedigrees_modified.pdf]
