# Supplementary material for: Novel TTLL5 Variants Associated with Cone-Rod Dystrophy and Early-Onset Severe Retinal Dystrophy
Source: Int J Mol Sci. 2021 Jun 15;22(12):6410. doi: 10.3390/ijms22126410 (PMC8232641; doi:10.3390/ijms22126410)
Supplement: Supplementary file 1 [file ijms-22-06410-s001.zip › Supplemental Figure S2_Conservation analysis_ckecked.pdf]

Supplemental Figure S2: Conservation analysis of the novel missense variants on *TTLL5*

Protein sequence alignment was performed using https://www.uniprot.org/align/. The Uniprot entry number is indicated on the left.

```
                                                                                p.(Trp492Arg) p.(Met505Val)
Q6EMB2      Homo sapiens               467       KVLRRVKEENDRRGGFIRIFPTSETWEIYGSYLEHKTSMNYMLATRLFQD----------   516
G1S3K9      Nomascus leucogenys        467       KVLRRVKEENDRRGGFIRIFPTSETWEIYGSYLEHKTSMNYMLATRLFQD----------   516
H2Q8N4      Pan troglodytes            467       KVLRRVKEENDRRGGFIRIFPTSETWEIYGSYLEHKTSMNYMLATRLFQDRGNPRRSLL-   525
A0A5F7ZXY3  Macaca mulatta             481       KVLRRVKEENDRRGGFIRIFPTSETWEIYGSYLEHKTSMNYMLATRLFQDRGNPRRSLL-   539
Q8CHB8      Mus musculus               467       KVLRRVKEENDRRGGFIRIFPTSETWEIYGSYLEHKTSMNYMLATRLFQDRGNPRRSLL-   525
D3ZAA1      Rattus norvegicus          369       KVLRRVKEENDRRGGFIRIFPTSETWEIYGSYLEHKTSMNYMLATRLFQDRGNTRRSLL-   427
F1PTL6      Canis lupus familiaris     467       KVLRRVKEENDRRGGFIRIFPTSETWEIYGSYLEHKTSMNYMLATRLFQDRGNPRRSLL-   525
M3WKA0      Felis catus                467       KVLRRVKEENDRRGGFIRIFPTSETWEIYGSYLEHKTSMNYMLATRLFPDRGNPRRSLL-   525
K7GRP4      Sus scrofa                 466       KVLRRVKEENDRRGGFIRIFPTSETWEIYGSYLEHKTSMNYMLATRLFQDR---------   516
E1BD55      Bos taurus                 438       KVLRRVKEENDRRGGFIRIFPTSETWEIYGSYLEHKTSMNYMLATRLFQDRWRFANLIN-   496
A0A1L1RZ47  Gallus gallus              482       KVLRRVRDENERRGGFIRIFPTPLTWDLYGSFLEYKTSMNYMLATRLFQDRDKMKGDLI-   540
F7ENG2      Xenopus tropicalis         455       KILRRVQDEYERRGGFVRIFPRHNTWQLYGSFLEYKTSLNYMLVTHLFPNRAAGNDH---   511
E9P886      Caenorhabditis elegans     543       QIVRKAELEDGRRGDFIRVFPRNGTWGMYSPVMEDLGNEDF--DERLFDEVVTKKNTKNS   600
Q9VBX5      Drosophila melanogaster    604       RILRNARLQYSRRGGFVRIFPTDDSMQRYGNFLDSANGIPISTPNVQSQ-----------   652
```
