# Supplementary material for: Novel TTLL5 Variants Associated with Cone-Rod Dystrophy and Early-Onset Severe Retinal Dystrophy
Source: Int J Mol Sci. 2021 Jun 15;22(12):6410. doi: 10.3390/ijms22126410 (PMC8232641; doi:10.3390/ijms22126410)
Supplement: Supplementary file 1 [file ijms-22-06410-s001.zip › Supplemental Figure S3_Large multiexons deletions IGV screenshot_modified.pdf]

TTLL5 gene

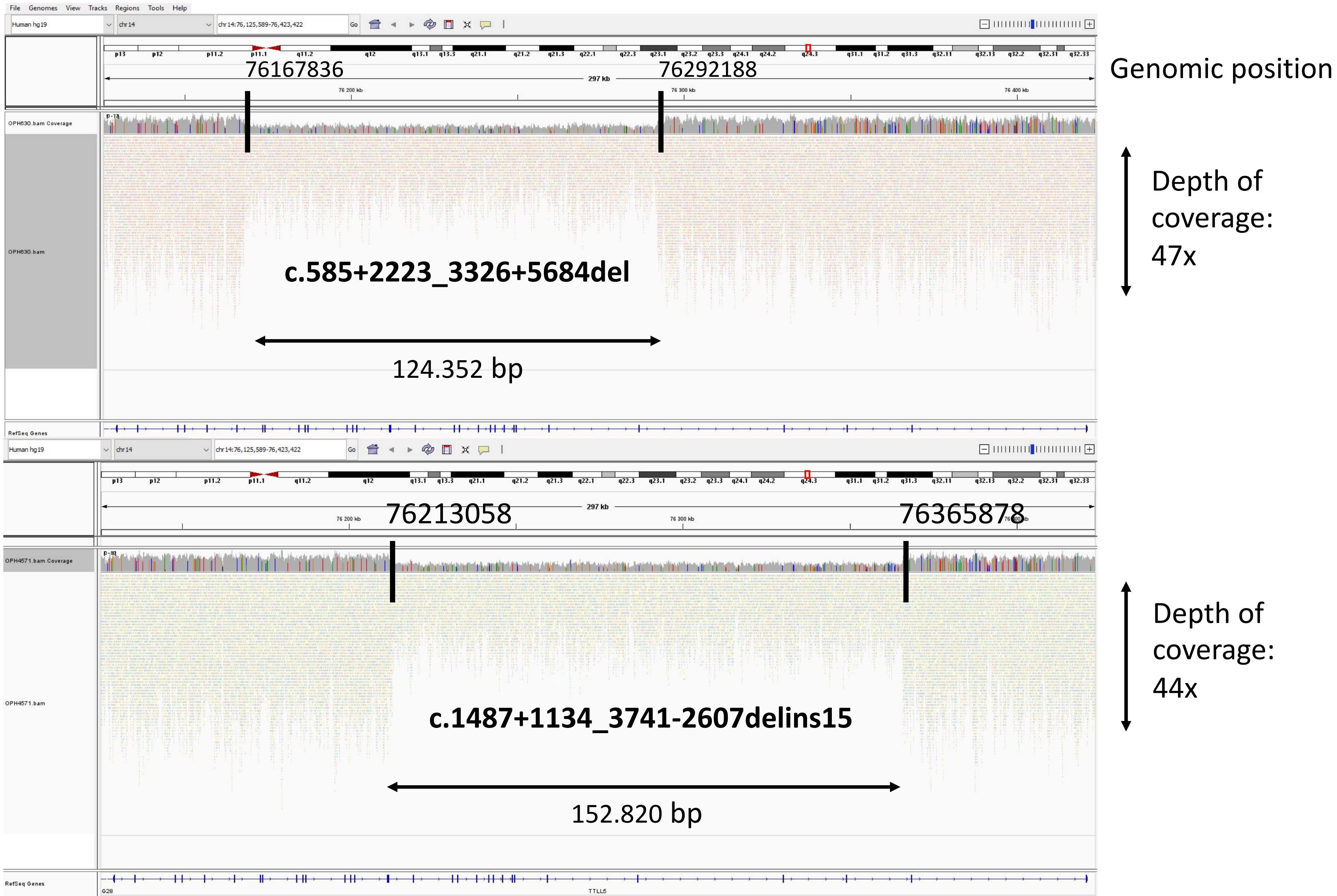
Genomic position
76167836
76292188
Depth of coverage: 47x
c.585+2223_3326+5684del
124.352 bp
76213058
76365878
Depth of coverage: 44x
c.1487+1134_3741-2607delins15
152.820 bp

Supplemental Figure S3: Large multiexon deletions depicted by IGV screenshots. Patients XZ-358338 and IM-4476 were heterozygous for large intragenic deletions, encompassing exons 8 to 28 and exons 18 to 30, respectively. Exact breakpoints were detected by whole genome sequencing followed by targeted *TTLL5* gene analysis. The mean depth of coverage out of the deletions was 47 and 44, respectively.
