# Supplementary material for: Novel TTLL5 Variants Associated with Cone-Rod Dystrophy and Early-Onset Severe Retinal Dystrophy
Source: Int J Mol Sci. 2021 Jun 15;22(12):6410. doi: 10.3390/ijms22126410 (PMC8232641; doi:10.3390/ijms22126410)
Supplement: Supplementary file 1 [file ijms-22-06410-s001.zip › Supplemental Figure S4_Breakpoint analysis_checked.pdf]

Supplemental Figure S4: Multiple sequence alignment and schematic overview of breakpoint analysis. **A.** In green, the LINE HAL1ME element, in blue the LTR gypsy element and in yellow the regions of microhomology. Oligo(G)n tracts are in bold underscored. **B.** In green, the SINE *AluJb* element, in blue the DNA Tigger16b element and in yellow the regions of microhomology. Inverted sequences are framed and A phased repeat is in bold.

A. c.585+2223_3326+5684del

```
Proximal     TTTCTACTGTGGTTGTGTAGGACTCTTTTCCTAATATCTTCTCCCTGGAAGAGGCTGACT
Junction     TTTCTACTGTGGTTGTGTAGGACTCTTTTCCTAATATCTTCTCCCTGGAAGAGGCTGACT
Distal       CCTCACCTTTCTCCTGAGTCTTAACTCTCACTATTCCTTCCCCTGAGGAAAACGATTT--

Proximal     GTGAATTTTGTGTAAGGGCAAAACATGTTGACTCTCAGGCTTTACTTTAGGTGGACATGG
Junction     GTGAATTTTGTGTAAGGGCAAAACATGTTGACTTCCATCTGAATTTTCTCTGCTACTTTT
Distal       CTCCAGTACATGTA--TGATAAAGGCCTGTACTTCCATCTGAATTTTCTCTGCTACTTTT

Proximal     TGAGAAGCACTCCCCTTCC-ACCTTGCCAAAATAAAGTGGGCCATATTGTGAATGCAGAG
Junction     CTCACTCAGCCAGACACGTAACCTCTCAAGAGTTATACGCTGCATCAGTTCAAGCTCTGT
Distal       CTCACTCAGCCAGACACGTAACCTCTCAAGAGTTATACGCTGCATCAGTTCAAGCTCTGT
```

B. c.1487+1134_3741-2607delins15

```
Proximal     TGCAACCTTGGCTTCCTGGGCTCCACTTCAAGCTATCCTGCCACCTCAGCCTGCCAGGTA
Junction     TGCAACCTTGGCTTCCTGGGCTCCACTTCAAGCTATCCTGCCACCTCAGCCTGCCAGGTA
Distal       TACCCATAATGTGCCTAAAGTAGTAATAACAGACCTAAACCATTTTGTGTACCAGTGTTT

Proximal     GCTAGGACTACAGGTACATGCC---------------------------ACTGTGCATGA
Junction     GCTAGGACTACAGGTACATGCCCCTTGGATTGTACCTGTTTCCCCTTGGATTGCTGTGGA
Distal       CCATGGAAAAATTCACTCAAAA---------------GTTTCCCCTTGGATTGCTGTGGA

Proximal     CTCATTTTTGTATTTTTTTGTAGAGATGGGGTCTCCCCATGTTGCCCAAGCTGATCTTGA
Junction     CACATTTCTACA--TCACAGGGAGAATGGGGACTCTATGCTAGAAGCAGTAGAATCATTA
Distal       CACATTTCTACA--TCACAGGGAGAATGGGGACTCTATGCTAGAAGCAGTAGAATCATTA
```
