# Supplementary material for: Novel TTLL5 Variants Associated with Cone-Rod Dystrophy and Early-Onset Severe Retinal Dystrophy
Source: Int J Mol Sci. 2021 Jun 15;22(12):6410. doi: 10.3390/ijms22126410 (PMC8232641; doi:10.3390/ijms22126410)
Supplement: Supplementary file 1 [file ijms-22-06410-s001.zip › Supplemental Table S1_List of genes tested.pdf]

Supplemental Table S1: List of genes composing the four NGS panels used

**Lille panel (156 IRD genes)**	**Lille panel (208 IRD genes)**	**Strasbourg panel (266 IRD genes)**	**Institut de la Vision panel (124 IRD genes)**
ABCA4	ABCA4	ABCA4	ABCA4
ADAM9	ABDH12	ABCC6	ADAM9
AIPL1	ADAM9	ABHD12	AIPL1
ARL2BP	ADAMTS18	ACBD5	ARL6
ARL6	ADIPOR1	ADAM9	ASTN2
BBS1	AGBL5	ADAMTS18	ATXN7
BBS2	AHI1	ADGRV1	BBS1
BBS12	AHR	AHI1	BBS10
BBS7	AIPL1	AIPL1	BBS12
BEST1	ALMS1	ALMS1	BBS2
C12orf65	ARHGEF18	AMACR	BBS4
C1QTNF5	ARL2BP	ANKS6	BBS5
C21orf2	ARL3	ARL13B	BBS7
C2orf71	ARL6	ARL2BP	BBS9
C2orf86	ASRGL1	ARL3	BEST1
C8orf37	ATF6	ARL6	C1QTNF5
CA4	ATXN7	ATXN7	C2orf71
CABP4	BBS1	B9D1	C8orf37
CACNA1F	BBS10	B9D2	CA4
CACNA2D4	BBS12	BBIP1	CABP4
CAPN5	BBS2	BBS1	CACNA2D4
CDH23	BEST1	BBS10	CC2D2A
CDH3	C12orf65	BBS12	CDH23
CDHR1	C1QTNF5	BBS2	CDHR1
CERKL	C21orf2	BBS4	CEP290
CEP290	C2orf71	BBS5	CERKL
CHM	C8orf37	BBS7	CHM
CLRN1	CA4	BBS9	CLN3
CNGA1	CABP4	BEST1	CLRN1
CNGA3	CACNA1F	C12orf65	CNGA1
CNGB1	CACNA2D4	C1QTNF5	CNGB1
CNGB3	CAPN5	C21orf2	CRB1
CNNM4	CC2D2A	C2orf71	CRX
CRB1	CCT2	C5orf42	CYP4V2
CRX	CDH3	C8orf37	DFNB31
CTNNA1	CDHR1	CA4	DHDDS
CYP4V2	CERKL	CABP4	EYS
DFNB31	CEP164	CACNA1F	FAM161A
DHDDS	CEP250	CACNA2D4	FLVCR1
DHX38	CEP78	CAPN5	FSCN2
DRAM2	CEP290	CC2D2A	GNPTG
EFEMP1	CFH	CCDC28B	GPR98

ELOVL4
EMC1
EYS
FAM161A
FLVCR1
FSCN2
FZD4
GNAT1
GNAT2
GRP125
GRP179
GRP98
GRK1
GRM6
GUCA1A
GUCA1B
GUCA2A
GUCY2D
HK1
IDH3B
IMPDH1
IMPG1
IMPG2
IQCB1
ITM2B
KCNJ13
KCNV2
KIAA1549
KIZ
KLHL7
LCA5
LRAT
LRIT3
LRP5
MAK
MERTK
MFRP
MFSD8
MVK
MYO7A
NDP
NEK2
NMNAT1
NR2E3
NRL
NYX

CHM
CLCC1
CLN3
CLRN1
CLUAP1
CNGA1
CNGA3
CNGB1
CNGB3
CNNM4
COL4A5
CRB1
CRX
CSPP1
CTNNA1
CYP4V2
DHDDS
DHX38
DRAM2
DTHD1
EFEMP1
ELOVL4
EMC1
EYS
FAM161A
FLVCR1
FSCN2
GDF6
GNAT1
GNAT2
GNB3
GRP125
GRP179
GRK1
GRM6
GUCA1A
GUCA1B
GUCY2D
HGSNAT
HK1
HMCN1
IDH3B
IFT140
IFT172
IFT81
IMPDH1

CDH23
CDH3
CDHR1
CEP164
CEP250
CEP290
CEP41
CEP83
CERKL
CHM
CIB2
CLN3
CLN5
CLN6
CLN8
CLRN1
CNGA1
CNGA3
CNGB1
CNGB3
CNNM4
CRB1
CRX
CSPP1
CTSD
CYP4V2
DCDC2
DFNB31
DHDDS
DHX38
DMD
DNAJC5
DRAM2
DTHD1
EFEMP1
ELOVL4
EMC1
ERCC1
ERCC2
ERCC3
ERCC4
ERCC5
ERCC6
ERCC8
EYS
FAM161A

GUCA1A
GUCA1B
GUCY2D
IDH3B
IMPDH1
IMPG2
INVS
IQCB1
KCNJ13
KLHL7
LCA5
LRAT
LZTFL1
MAK
MERTK
MFRP
MKKS
MKS1
MYO7A
NMNAT1
NPHP1
NPHP3
NPHP4
NR2E3
NRL
OAT
OFD1
OTX2
PAF1
PANK2
PCDH15
PDE6A
PDE6B
PDE6C
PDE6G
PDZD7
PEX1
PEX7
PHYH
PITPNM3
PRCD
PROM1
PRPF3
PRPF31
PRPF6
PRPF8

OAT
OFD1
OPN1LW
OPN1MW
OPN1SW
OTX2
PCDH15
PDE6A
PDE6B
PDE6C
PDE6G
PDE6H
PDZD7
PGK1
PITPNM3
PLA2G5
PRCD
PROM1
PRPF3
PRPF31
PRPF4
PRPF6
PRPF8
PRPH2
RAB28
RAX2
RB1
RBP3
RBP4
RD3
RDH12
RDH5
RGR
RGS9
RGS9BP
RHO
RIMS1
RLBP1
ROM1
RP1
RP1L1
RP2
RP9
RPE65
RPGRP
RPGRIP1

IMPG1
IMPG2
INPP5E
IQCB1
ITM2B
JAG1
KCNJ13
KCNV2
KIAA1549
KIF11
KIZ
KLHL7
LAMA1
LCA5
LRAT
LRIT3
LRP5
MAK
MAPKPK3
MERTK
MFRP
MFSD8
MVK
NDP
NEK2
NEUROD1
NMNAT1
NPHP1
NPHP10
NPHP2
NPHP3
NPHP4
NR2E3
NRL
NYX
OAT
OFD1
OPN1LW
OPN1MW
OPN1SW
OTX2
PDE6A
PDE6B
PDE6C
PDE6G
PDE6H

FIZ1
FLVCR1
FSCN2
FZD4
GDF6
GLIS2
GNAT1
GNAT2
GNPTG
GPR125
GPR179
GRK1
GRM6
GRN
GUCA1A
GUCA1B
GUCY2D
HARS
HGSNAT
HK1
HMX1
IDH3B
IFT122
IFT140
IFT172
IFT27
IFT80
IFT88
IMPDH1
IMPG1
IMPG2
INPP5E
INVS
IQCB1
ITM2B
KCNJ13
KCNV2
KIAA1549
KIF11
KIF5A
KIF7
KIZ
KLHL7
LAMA1
LCA5
LRAT

PRPH2
RAX2
RBP3
RBP4
RD3
RDH12
RDH5
RGR
RHO
RIMS1
RLBP1
ROM1
RP1
RP2
RP9
RPE65
RPGR
RPGRIP1
RPGRIP1L
SAG
SEMA4A
SNRNP200
SPATA7
TMEM237
TOPORS
TRIM32
TTC8
TTLL5
TTPA
TULP1
UNC119
USH1C
USH1G
USH2A
WDPCP
ZNF513

RS1
SAG
SEMA4A
SLC24A1
SLC7A14
SNRNP200
SPATA7
TEAD1
TIMP3
TMEM67
TOPORS
TRIM32
TRPM1
TSPAN12
TTC8
TTLL5
TULP1
UNC119
USH1C
USH2A
VCAN
ZNF513

PDZD7
PEX1
PEX6
PEX26
PEX7
PGK1
PHYH
PITPNM3
PLA2G5
POC1B
POMGNT1
PRCD
PRDM13
PROM1
PRPF3
PRPF31
PRPF4
PRPF6
PRPF8
PRPH2
PYGM
RAB28
RAX2
RBP3
RBP4
RCBTB1
RD3
RDH11
RDH12
RDH5
REEP6
RGR
RGS9
RGS9BP
RHO
RIMS1
RLBP1
ROM1
RP1
RP1L1
RP2
RP9
RPE65
RPGRP
RPGRIP1
RS1

LRIT3
LRP5
LZTFL1
MAK
MERTK
MFRP
MFSD8
MIR204
MKKS
MKS1
MVK
MYO7A
NDP
NEK2
NEK8
NEUROD1
NMNAT1
NPHP1
NPHP3
NPHP4
NR2E3
NR2F1
NRL
NYX
OAT
OFD1
OPN1LW
OPN1MW
OPN1SW
OR2W3
OTX2
PANK2
PAX2
PCDH15
PCYT1A
PDE6A
PDE6B
PDE6C
PDE6D
PDE6G
PDE6H
PDZD7
PEX1
PEX10
PEX14
PEX16

SAG
SAMD11
SEMA4A
SLC24A1
SLC7A14
SNRNP200
SPATA7
SPP2
TIMP3
TMEM216
TOPORS
TRNT1
TRPM1
TSPAN12
TTC8
TTLL5
TUB
TUBGCP4
TUBGCP6
TULP1
UNC119
USH2A
VCAN
VPS13B
WDR19
ZNF408
ZNF423
ZNF513

PEX19
PEX2
PEX5
PEX7
PGK1
PHYH
PITPNM3
PLA2G5
PLK4
PNPLA6
POC1B
PPT1
PRCD
PROM1
PRPF3
PRPF31
PRPF4
PRPF6
PRPF8
PRPH2
RAB28
RBP3
RBP4
RD3
RDH11
RDH12
RDH5
RGR
RGS9
RGS9BP
RHO
RIMS1
RLBP1
ROM1
RP1
RP1L1
RP2
RP9
RPE65
RPGR
RPGRIP1
RPGRIP1L
RS1
SAG
SDCCAG8
SEMA4A

SLC19A2
SLC24A1
SLC7A14
SNRNP200
SPATA7
SRPX
TCTN1
TCTN2
TCTN3
TIMP3
TMEM216
TMEM231
TMEM237
TMEM238
TMEM67
TOPORS
TRIM32
TRPM1
TSPAN12
TTC21B
TTC8
TTLL5
TTPA
TUB
TUBGCP4
TULP1
UNC119
USH1C
USH1G
USH2A
VCAN
VPS13B
WDPCP
WDR19
WDR34
WDR35
XPNPEP3
ZNF408
ZNF423
ZNF513
